# Extra-articular Synovial Chondromatosis of the Subacromial-Subdeltoid Bursa Mimicking Rice Body Synovitis: A Case Report

**DOI:** 10.7759/cureus.116562

**Published:** 2026-09-19

**Authors:** Rathod Megharaj, Sherasha F Kammar, Vinay Pawar, Chandan G Naik

**Affiliations:** 1 Orthopedics, Karnataka Medical College and Research Institute, Hubballi, IND

**Keywords:** extra-articular synovial chondromatosis, rice body, shoulder pathology, subacromial-subdeltoid bursa, subacromial-subdeltoid bursitis

## Abstract

Synovial chondromatosis can lead to proliferation of the synovium followed by the appearance of multiple cartilaginous nodules, most commonly in large joints. Extra-articular involvement of the subacromial-subdeltoid bursa could resemble an inflammatory or infectious process, especially if the chondromatosis is present in the form of rice bodies. A 37-year-old right-hand-dominant male presented with left shoulder pain for three months, with restricted shoulder motion and pain during overhead activities, with no past medical history of trauma or surgery. He presented to the orthopedic outpatient department. Blood tests, including inflammatory markers, rheumatoid factor, and tuberculosis investigations, were normal. X-rays were unremarkable, but magnetic resonance imaging demonstrated enlargement of the bursa with multiple nodular loose bodies resembling rice bodies. Open excision revealed over 100 loose bodies measuring 3-10 mm. Synovectomy was performed, and histopathology demonstrated the presence of hyaline cartilage, confirming the diagnosis of synovial chondromatosis. The postoperative phase was uneventful. The patient underwent rehabilitation and demonstrated improved pain relief and shoulder function. At the six-month follow-up, he had improved shoulder range of motion and was free from pain, swelling, or clinical recurrence. This case highlights that extra-articular synovial chondromatosis can closely resemble rice body synovitis, making magnetic resonance imaging valuable for preoperative assessment, while histopathological examination remains essential for definitive diagnosis.

## Introduction

Synovial chondromatosis is a rare, benign disease that results in the formation of cartilaginous nodules within the synovium, which may become detached and lead to the formation of loose bodies. The disease is commonly associated with large joints like the knee, hip, or elbow, with shoulder involvement being rare. Extra-articular synovial chondromatosis, particularly in the subacromial-subdeltoid bursa, is infrequent [1,2].

Patients present with symptoms like chronic pain, swelling, and limitation in movements, along with other symptoms that may lead to confusion in diagnosing subacromial bursitis, impingement, rotator cuff pathology, or inflammatory arthritis. It may be difficult to diagnose synovial chondromatosis with X-rays, especially when the cartilaginous nodules are not calcified. This is where magnetic resonance imaging (MRI) becomes useful in helping identify swollen bursal tissues and shows the characteristic nodular loose bodies [3,4].

Rice bodies are small, smooth, fibrinous bodies that typically develop in chronic inflammatory conditions such as rheumatoid arthritis and tuberculous synovitis. Because they can closely resemble the loose bodies of synovial chondromatosis on imaging, distinguishing between these entities is clinically important, as their underlying pathology and management differ substantially. Histopathological examination confirms the diagnosis and differentiates it from other causes of chronic synovitis and multiple loose bodies. The existence of a substantial number of loose bodies that resemble rice bodies may pose a further diagnostic challenge due to the frequent association of rice bodies with rheumatoid arthritis and tuberculosis. The treatment of choice would be complete excision of the affected synovial membrane, along with resection of loose bodies [5,6].

This report describes a case of extensive extra-articular synovial chondromatosis of the subacromial-subdeltoid bursa with over 100 rice body-like loose bodies, and with histopathological confirmation of chronic synovitis with chondromatosis. This case is unusual because it involved isolated subacromial-subdeltoid bursal disease with over 100 rice body-like loose bodies, closely mimicking inflammatory or tuberculous bursitis despite the absence of systemic inflammatory disease, thereby highlighting the potential for significant preoperative diagnostic confusion.

## Case presentation

A 37-year-old right-hand-dominant male presented with complaints of gradual left shoulder pain lasting for three months. The pain was getting worse, along with limited shoulder movement. He experienced pain while performing overhead tasks. There was no history of trauma or injury. He did not complain of any systemic symptoms such as fever, weight loss, or night sweats. He had no history of tuberculosis, rheumatoid arthritis, other inflammatory joint disorders, past shoulder surgeries, or local corticosteroid injections.

During clinical examination, the patient appeared comfortable at rest. His left shoulder had mild tenderness and restriction of motion. Active and passive ranges of motion were restricted due to pain during forward elevation and abduction. External and internal rotation were less painful than forward elevation and abduction. Active forward flexion was 120°, abduction was 90°, external rotation was 45°, and internal rotation was limited to the L3 vertebral level. These clinical findings were correlated with the normal plain radiographic findings on the anteroposterior shoulder X-ray, which showed no significant osseous abnormalities (Figure 1). Mild crepitus could be felt at the shoulder. There was no visible abnormality or instability in the shoulder. The strength of the rotator cuff appeared to be preserved, and there were no signs of a large rotator cuff tear.

**Figure 1:**
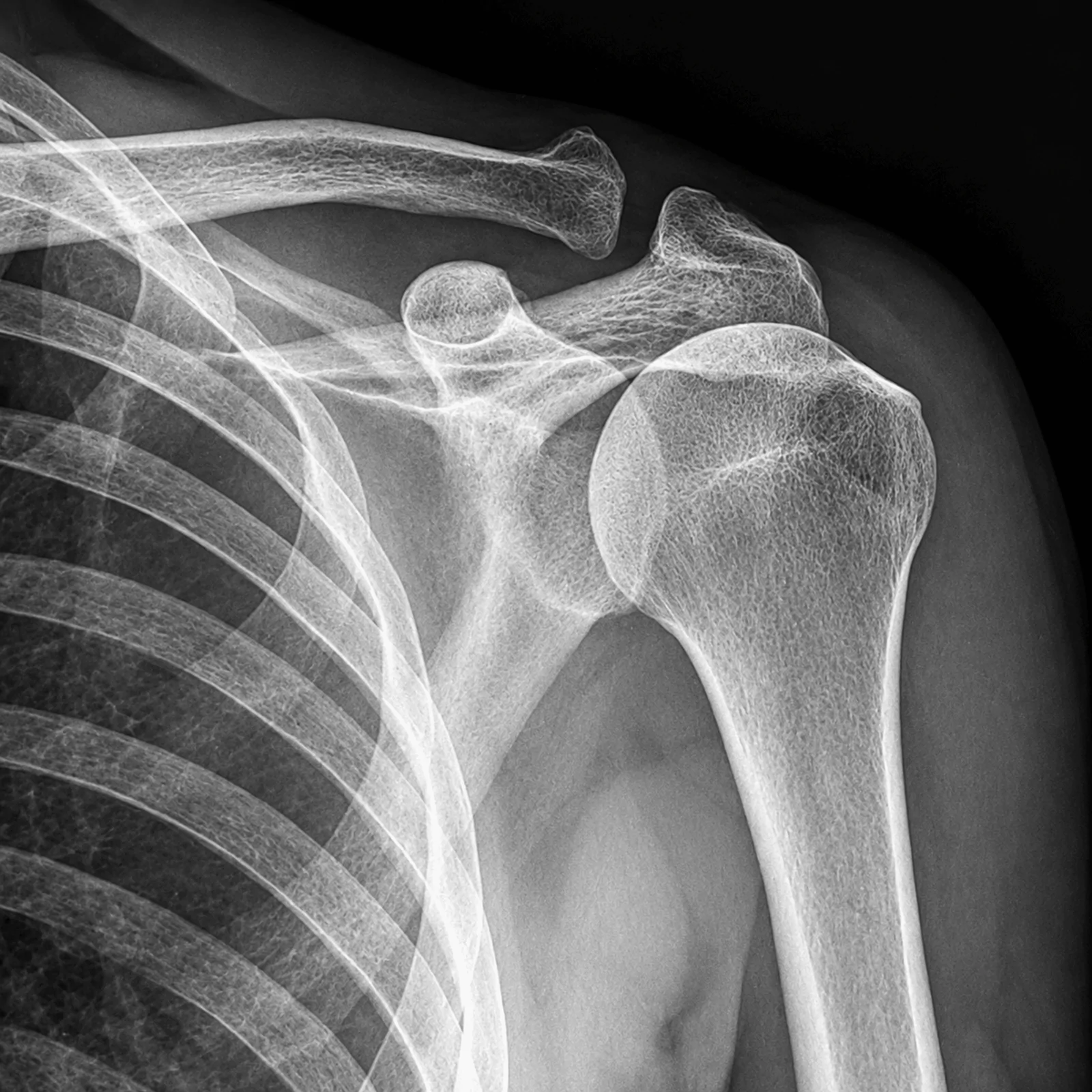
Preoperative X-ray of the left shoulder (anteroposterior view). The radiograph demonstrates preserved glenohumeral alignment with no visible calcified loose bodies, osseous erosion or destruction, fracture, or significant degenerative changes.

Baseline laboratory tests, including a complete blood count, erythrocyte sedimentation rate, C-reactive protein, and rheumatoid factor, were within normal limits (Table 1). Tuberculosis evaluation, including a Mantoux test and chest radiography, showed no evidence of active or previous tuberculosis. There was no sign of systemic infection or active inflammatory disease. Given the recurrent symptoms, the decision was taken to conduct an MRI of the left shoulder.

**Table 1:** Baseline laboratory investigations showing normal hematological and inflammatory parameters without evidence of systemic infection or active inflammatory disease.

Laboratory investigation	Patient value	Reference range
Hemoglobin (g/dL)	13.8	12.0-16.0
White blood cell count (×10³/µL)	7.2	4.0-11.0
Platelet count (×10³/µL)	286	150-450
Erythrocyte sedimentation rate (mm/h)	8.0	0-20.0
C-reactive protein (mg/L)	1.8	<5.0
Rheumatoid factor (IU/mL)	7.0	<14.0

MRI demonstrated marked distension of the subacromial-subdeltoid bursa containing numerous T2 hyperintense, well-circumscribed nodular loose bodies measuring 3-10 mm, associated with diffuse synovial thickening without glenohumeral joint involvement (Figures 2A-2C). Diagnostic imaging characteristics of the lesions were compatible with cartilaginous loose bodies. The presence of significant synovial thickening and diffuse bursal involvement was noted. The glenohumeral joint surfaces were preserved, and there was no active intra-articular disease. No aggressive marrow infiltration or destructive bone pathology was observed. The rotator cuff was intact, with no considerable tear. The imaging results pointed to the probable diagnosis of synovial chondromatosis affecting the subacromial and subdeltoid bursa.

**Figure 2:**
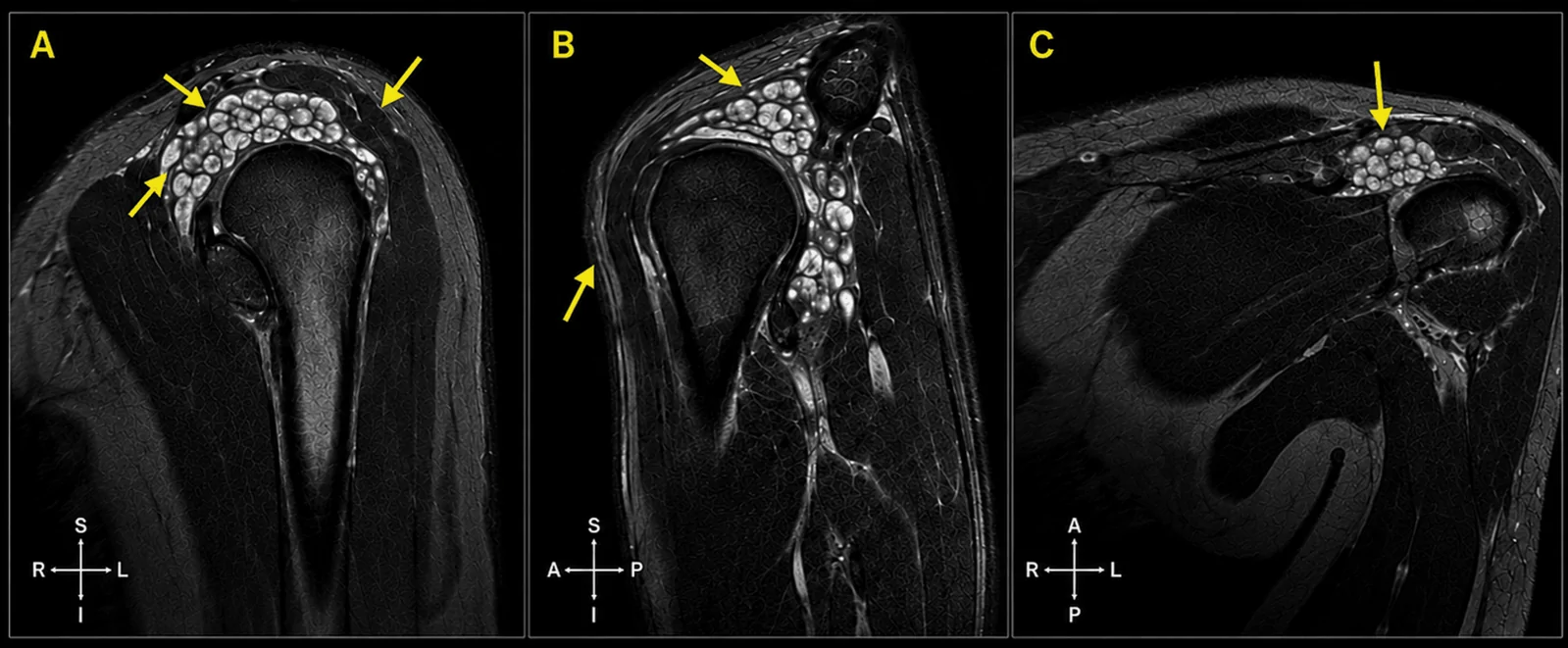
Preoperative magnetic resonance imaging of the left shoulder. (A) Coronal image showing marked distension of the subacromial-subdeltoid bursa containing multiple well-defined T2 hyperintense nodular loose bodies (yellow arrows). (B) Sagittal image demonstrating a distended bursa with numerous loose bodies (yellow arrows) and diffuse synovial thickening of the bursal wall. (C) Axial image revealing multiple loose bodies (yellow arrows) surrounding the humeral head within the subacromial-subdeltoid bursa. The glenohumeral joint is preserved without intra-articular loose bodies.

The differential diagnosis included the presence of chronic inflammatory bursitis, tuberculosis bursitis, rheumatoid bursitis, pigmented villonodular synovitis, and soft tissue chondroma. The large number of well-defined cartilaginous nodules and their specific MRI appearance supported the diagnosis of synovial chondromatosis.

Considering the serious disease burden, persistent symptoms, and considerable limitation in shoulder function, surgery was suggested. An arthroscopic procedure was thought to be an option; however, due to the high number of loose bodies, significant bursal swelling, extensive synovial damage, and a wide, thickened false capsule, open surgery was preferred in order to ensure the complete removal of the loose bodies and proper removal of the inflamed synovial tissue.

A deltoid-splitting approach was used. The distended bursa was enclosed by a thickened pseudocapsule. Over 100 pearly-white loose bodies measuring 3-10 mm were removed, followed by complete bursectomy and synovectomy. The bodies had a very typical appearance, similar to rice bodies macroscopically. There were numerous shiny cartilage nodules found in the bursal cavity. There was an intraoperative inspection of the glenohumeral joint that showed no signs of involvement, meaning that the disease was mostly extra-articular.

The expanded bursa and affected pseudocapsule were completely removed, in addition to the extraction of all loose bodies that could be found. A complete synovectomy was performed on the affected bursal tissue. The removed tissue comprised several smooth, oval, pearlescent loose bodies, in addition to abundant fibrous and synovial tissue. The excised tissue and the loose bodies were sent for histopathology.

On gross examination, the tissues appeared as grayish-white lumps, grayish-brown lumps, or grayish-yellow lumps. Histological examination demonstrated mature hyaline cartilage nodules within chronically inflamed synovial tissue lined by hyperplastic synoviocytes with lymphoplasmacytic infiltration. No cytological atypia or malignant transformation was identified (Figures 3A, 3B). Once the histopathology report indicated chronic synovitis with chondromatosis, the diagnosis of synovial chondromatosis was confirmed. The presence of mature hyaline cartilage played an important role in confirming the cartilaginous nature of these rice body-like loose bodies.

**Figure 3:**
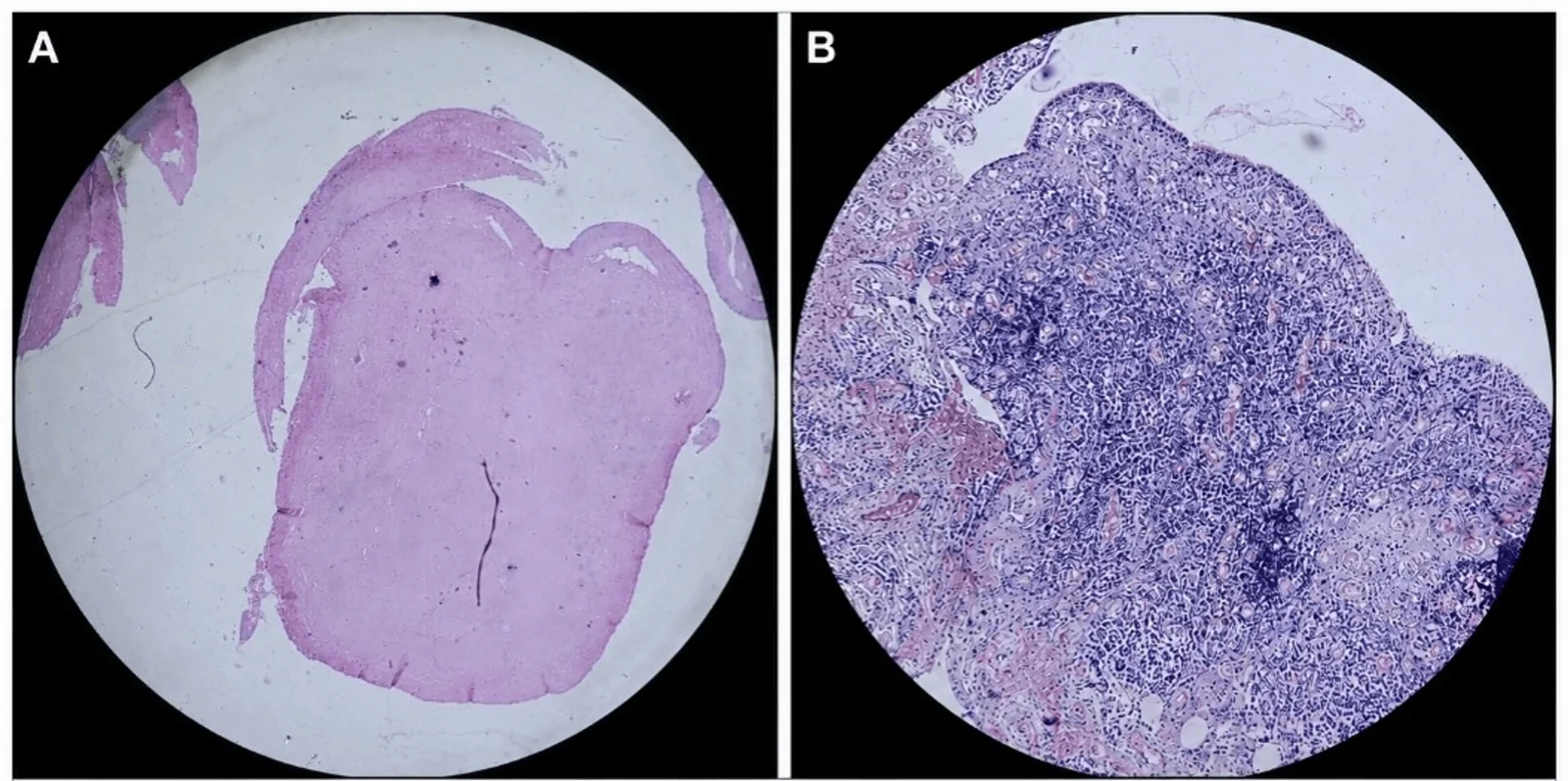
Histopathological examination of the excised specimen. (A) Low-power view showing a fragment of mature hyaline cartilage consistent with a loose body. (B) High-power view showing fibrocollagenous synovial tissue lined by synoviocytes with dense chronic inflammatory infiltrates composed predominantly of lymphocytes and plasma cells, consistent with chronic synovitis with chondromatosis.

Postoperative management involved the use of analgesics, wound care, and a structured rehabilitation program. The rehabilitation program included early shoulder mobilization, followed by gradual progressive active and passive range of motion exercises, accompanied by strengthening exercises depending on the tolerance for pain and the healing of soft tissues.

Post-surgery, the patient continued to experience a reduction in pain and improvement in shoulder function and mobility. At the six-month follow-up, the patient reported marked pain relief and return to routine daily activities. Active forward flexion improved from 120° to 170°, abduction from 90° to 170°, external rotation from 45° to 80°, and internal rotation improved from the L3 to the T8 vertebral level. There was no pain, swelling, crepitus, or clinical evidence of recurrence.

## Discussion

Synovial chondromatosis is a rare, benign condition that is characterized by the formation of multiple nodules due to synovial metaplasia. The condition generally involves large joints, while the shoulder joint is most often unaffected. It has been reported that isolated cases involving the subacromial-subdeltoid bursa are extremely rare but may lead to considerable symptoms and impairment of shoulder movement [2,3].

Clinical diagnosis is not straightforward, as the shoulder pain due to the bursa being impacted may appear similar to other conditions, including subacromial bursitis, rotator cuff syndrome, or inflammation. MRI is a helpful tool; in many cases, cartilaginous nodules could be undetectable on X-rays [1].

The resemblance of loose bodies to rice bodies is significant in that it can cause diagnostic errors. The presence of rice bodies was defined by Rush et al. as indicative of tissue inflammation of a long duration in chronic arthritis, typically rheumatoid arthritis or tuberculous arthritis [7]. The term rice body does not apply to loose bodies formed by synovial chondromatosis, as the latter type of loose bodies is easy to recognize due to their composition of calcified or non-calcified cartilage [8]. Cao et al. presented a similar case of rice bodies in the shoulder in a patient suffering from longstanding instances of rheumatoid arthritis, wherein histopathological analysis revealed soft necrotic tissue surrounded by fibrin without cartilage or chondromas [6]. Unlike the classical rice body appearance in previous cases, this case presented loose bodies containing hyaline cartilage inside them. Complete synovectomy is considered important because residual metaplastic synovium may contribute to recurrence.

Notably, Cheung et al. established that MRI characteristics of rice body-like particles are not specific for true rice bodies. They reported that synovial chondromatosis caused the appearance of rice body-like particles on MRI in some cases, and some lesions that were shown to resemble rice bodies were later confirmed to have synovial chondromatosis through histology [8]. This finding is significant in this case, in which the appearance was similar to rice bodies, but the results of histopathology proved that the real tissue type was mature hyaline cartilage. Therefore, the term “rice body-like” seems to be more appropriate for the description of these lesions prior to any histological confirmation.

The significance of extra-articular distribution of loose bodies lies in the fact that there may be extensive disease in the subacromial-subdeltoid bursa without much involvement of the glenohumeral joint. The extra-articular bursal cavity may experience a great accumulation of loose bodies in an enclosed space, thereby producing progressive bursa expansion and inflammatory changes to the synovium, which leads to significant limitation of movement, despite the articular structures not being affected to such an extent [6]. Li et al. suggested that extra-articular loose bodies in the subacromial space may originate from proliferative synovial bursal tissue, something that was found important in connection with the pathogenesis of isolated bursal synovial chondromatosis [9].

## Conclusions

Subacromial-subdeltoid bursa extra-articular synovial chondromatosis is a rare condition that can result in shoulder pain and limitation of motion. The presence of rice body-like loose bodies can lead to confusion in diagnosis since they can resemble tuberculous or inflammatory bursitis. Imaging findings on MRI are useful in assessing the size of the bursa, while histopathology confirms the condition. Complete synovectomy with loose-body removal was associated with symptomatic and functional improvement in this patient. At the six-month follow-up, there was no clinical evidence of recurrence.
